# Wood biochar enhances methanogenesis in the anaerobic digestion of chicken manure under ammonia inhibition conditions

**DOI:** 10.1016/j.heliyon.2023.e21100

**Published:** 2023-10-21

**Authors:** Tien Ngo, Leadin S. Khudur, Christian Krohn, Soulayma Hassan, Kraiwut Jansriphibul, Ibrahim Gbolahan Hakeem, Kalpit Shah, Aravind Surapaneni, Andrew S. Ball

**Affiliations:** a School of Science, RMIT University, Melbourne, VIC 3083, Australia; b ARC Training Centre for the Transformation of Australia's Biosolids Resource, RMIT University, Bundoora, VIC 3083, Australia; c School of Engineering, RMIT University, Melbourne, VIC 3000, Australia; d South East Water, 101 Wells Street, Frankston, VIC 3199, Australia

**Keywords:** Chicken manure, Biochar, Biomethane, Methanogens, Methanogenic pathway, Ammonia inhibition

## Abstract

The process of breaking down chicken manure through anaerobic digestion is an effective waste management technology. However, chicken manure can be a challenging feedstock, causing ammonia stress and digester instability. This study examined the impacts of adding wood biochar and acid-alkali-treated wood biochar to anaerobically digest chicken manure under conditions of ammonia inhibition. The results highlighted that only the addition of 5 % acid-alkali-treated wood biochar by volume can achieve cumulative methane production close to the typical methane potential range of chicken manure. The treated wood biochar also exhibited highest total ammonia nitrogen removal compared to the Control treatment. Scanning Electron Microscope revealed growing interactions between biochar and methanogens over time. Real-time polymerase chain reaction showed that treated wood biochar produced the highest number of bacterial biomass. In addition, 16S amplicon-based sequencing identified a more robust archaeal community from treated biochar addition. Overall, the acid-alkali treatment of biochar represents an effective method of modifying biochar to improve its performance in anaerobic digestion.

## Graphical abstract

**Figure:**
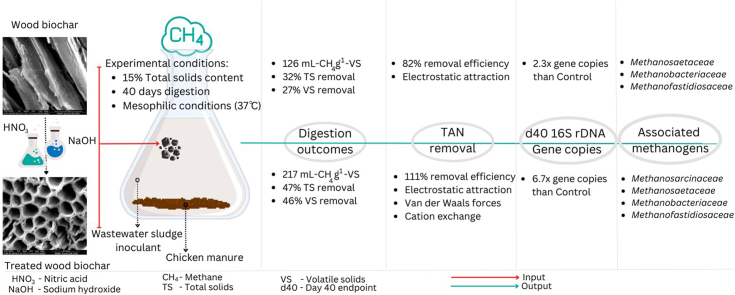

## 1 Introduction

In 2022, there were an estimated 33 billion chickens worldwide, which has increased 130 % since 2000 [1]. In recent years, chicken has become the most-produced meat in the world [1]. As chicken production has increased, so has the generation of chicken manure (CM). In Australia alone, over 1 million tonnes are produced per annum Wiedemann [2]. Chicken manure is the primary by-product of chicken meat production and is mainly composed of faeces and bedding materials such as sawdust, shavings, rice hulls, or straw [2].

While CM has been used primarily as a fertiliser, its direct land application can lead to various environmental concerns, such as eutrophication, pathogen contamination, air pollution, and emission of greenhouse gases [3]. Therefore, CM must generally undergo pretreatment or recycling before land disposal in order to minimise environmental impacts [[3], [4], [5]]. However, the high biodegradability and abundance of organic matter in CM make it a potential feedstock for anaerobic digestion (AD), which constitutes a process where microbes break down organic waste such as CM to generate a digestate with agricultural value and produce a source of renewable energy, methane (CH_4_) [3,5]. However, establishing optimal conditions to digest CM anaerobically remains an ongoing challenge. Chicken manure has a high nitrogen content, between 3.4 % and 3.9 %, by dry weight [6]. The nitrogen arises from two sources: up to 70 % from uric acid and the remaining 30 % from undigested proteins [6,7]. During AD, these nitrogenous compounds are biologically converted to ammonia (NH_3_), also known as Free Ammonia Nitrogen (FAN), and ammonium ions (NH_4_^+^); together they account for the Total Ammonia Nitrogen (TAN) [7,8]. As NH_3_ accumulates, toxicity toward methanogens increases as more NH_3_ diffuses across methanogenic cell membranes [7,8]. A recent review by Cai, Zheng [9] reported that a TAN concentration of over 2000 mg L^−1^ can negatively impact CH_4_ production efficiency. Ammonia toxicity can also be temperature and pH dependent, as higher temperature and pH will result in more FAN formation [9]. Furthermore, in a detailed study on ammonia inhibition by Rajagopal, Massé [10], it was established that Total Ammonia Nitrogen (TAN) concentration exceeding 3000 mg L^−1^ will result in total inhibition at any pH.

To alleviate ammonia inhibition, several methods have been successfully utilised such as ammonia stripping. Ammonia stripping is a prevalent method to manage ammonia toxicity; its widespread adoption can be attributed to its simplicity, cost-effectiveness and scalability [11]. For digesters with high TAN concentrations, such as those treating CM, the elevated pH levels can diminish the bioavailability of trace elements. (TE). Trace elements supplementation can be employed to increase their bioavailability and improve digester efficiency; trace elements are essential for synthesis of metalloenzymes [12]. Another novel strategy to mitigate ammonia inhibition is bioaugmentation. A recent study by Wang, Wang [13] successfully employed bioaugmentation to stabilise ammonia inhibited AD systems treating CM. Other methods such as bentonite addition, co-digestion, water extraction, and pH adjustment have also been reported in the scientific literature [[14], [15], [16], [17]].

Recently, biochar addition to AD systems treating nitrogen-rich feedstock has been a promising strategy to alleviate ammonia inhibtion. Biochar is a material composed of carbon synthesised via thermal chemical transformation processes using various biomass such as agricultural residue, forestry byproducts, sewage slurry, and solid waste from municipalities. The various physical and chemical properties of biochar, including ample porosity and large surface area for microbial acclimitisation, high absorptive capacity and cation exchange capacity for NH_4_^+^, essential TE components for supplementation, effective pH buffer, and surface functional groups for adsorption of inhibitory compounds, have proven to be effective in the alleviation of NH_3_ stress [4,18,19]. When compared to the above-mentioned methods which are monofaceted, a simple biochar addition can offer a multifaceted approach to mitigate ammonia stress. Biochar addition can reduce TAN concentration via adsorption and concurrently, provide anchorage point for microbial growth, buffer against unfavourable pH and supply essential trace elements.

Biochar can also be further treated using acid, alkali, or both to improve its surface properties. For example, in a previous study, the efficacy of wood biochar and its acid-alkali-treated variant in alleviating NH_3_ stress was explored [5]. Acid-alkali-treated biochar (TBC) improved CH_4_ production by 77.5 % compared to no biochar controls [5]. Acid-alkali-treated biochar also demonstrated the highest rate of TAN removal; TBC was concluded to have a higher adsorption capacity towards NH_4_^+^ than its untreated version [5]. In an NH_4_^+^ adsorption study by Vu, Trinh [20], acid-alkali biochar treatment produced approximately six times improvement in the maximum capacity of adsorption for NH_4_^+^, from 3.93 mg g^−1^ to 22.6 mg g^−1^, calculated using the Langmuir isotherm.

In this study, the interactions between wood biochar and its treated variant with the methanogenic population were explored. While the current literature suggests that *Methanosarcinaceae* is more likely to dominate manure digesters [21], it is hypothesised that the presence of biochar may allow for a more diverse archaeal community to thrive under NH_3_ stress. Furthermore, this study aims to investigate whether the properties arising from acid-alkali treatment of biochar will improve the resilience of the methanogenic population towards ammonia stress. These improvements are hypothesised to be in the form of enhanced microbial attachment and sheltering, as well as increase in TAN adsorption. To the extent known by the authors, this is the first study that closely investigates acid-alkali treated biochar and its untreated version on their performance in an ammonia inhibited AD. In addition, the results of this investigation will also highlight the possible application and novelty of TBC as a cost-effective recovery agent for ammonia inhibited digesters.

## 2 Methodology

### 2.1 Characterisation of chicken manure and wastewater sludge

Chicken manure (CM) was retrieved from Bellarine Worms, situated in Point Lonsdale, Victoria, Australia. The collection process involved using trays to gather CM from broiler chickens, which was subsequently air-dried and packaged into bags. As for the unacclimated methanogenic inoculant, it was derived from thickened waste-activated sludge (TWAS), obtained from the Mount Martha municipal wastewater recycling plant operated by South East Water Corporation in Melbourne, Australia. The inoculant and feedstock were brought to RMIT University, Melbourne, Australia, and maintained at a temperature of 4 °C until they were ready for subsequent use. The CM (used as feedstock) underwent sieving to achieve a particle size range of 0.5–2 mm before digestion, while the inoculant was utilised directly without any prior treatment.

The CM, inoculant, and a combination of both underwent a thorough assessment of their physical and chemical attributes before commencing the digestion process. These measurements were carried out in triplicate. Parameters such as chemical oxygen demand (COD), electrical conductivity (EC), pH, and salinity were determined using samples mixed at a 1:10 weight-to-volume ratio with Milli-Q water. The COD concentrations were quantified using COD digestion vials and the HACH-DRB 200 (Loveland, Colorado, USA). Additionally, COD values from the processed samples were obtained by a HACH DR 900 colorimeter (Loveland, Colorado, USA) [5,22]. Degree of salinity and level of EC were assessed using a Compact Conductivity Meter, the LAQUAtwin-EC-11, and a Compact Salt Meter, the LAQUAtwin-Salt-11, both from HORIBA Scientific. To determine pH values, a pH probe, the HANNA Instrument edge^pH^ (Keysborough, Victoria, Australia) was employed. A 1:80 *weight-to-volume* ratio of digestates to Milli-Q water was used for TAN, total nitrogen (TN), nitrate (NO_3_^−^) and nitrite (NO_2_^−^) testing. For the determination of TAN, the salicylate method was applied. This method entailed the addition of prepared samples to a reagent, followed by the sequential addition of ammonium salicylate and ammonium cyanurate. To calculate Total Kjeldahl Nitrogen (TKN), the levels of nitrate (NO^3−^) and nitrite (NO^2−^) were subtracted from the total nitrogen (TN). Total nitrogen was determined using the persulfate digestion method, via a HACH Pacific Total Nitrogen Reagent set (Loveland, Colorado, USA). Nitrate was determined using the chromotropic acid method using the HACH Pacific NitraVer kit (Loveland, Colorado, USA), while nitrite was determined using the diazotisation method using the HACH Pacific NitriVer kit (Loveland, Colorado, USA). The analysis of the mixture for TAN, TKN, nitrate, and nitrite was performed using a HACH DR 900 colorimeter. Free Ammonia Nitrogen (FAN) was calculated using the following equation, as describe by Guo, Li [3]:

1] [FAN] = [TAN] / [10^(p*K*a−pH)^ +

pKa = $\frac{2729.92}{273+T}$, where T is 37 °C, the temperature of AD.

To determine Total Solids (TSs), a 10 g sample underwent heating in a 105 °C oven for 24 h. After a brief cooling period, the sample's weight was measured to obtain the TSs value. Volatile Solids (VSs) were assessed by subjecting oven-dried products from the previous step to 2 h of heating at 550 °C. Following a cooling process, the difference between the final weight and the initial weight is calculated as the VSs. Table 1 displays the physicochemical properties of the inoculant, chicken manure (CM), and a combination of both.

**Table 1:** Characteristics of feedstock (CM), wastewater sludge inoculant, and combination of both, before the commencement of anaerobic digestion, along with their respective units.

Characteristics		Feedstock (CM)	Inoculant	Feedstock (CM) and Inoculant
Chemical oxygen demand (COD)	g L^−1^	61.5 ± 0.35^a^	3.53 ± 0.23	10.7 ± 0.62
Total solids (TSs)^c^	%	80.6 ± 1.50	2.2 ± 0.00	14.6 ± 0.76
Volatile solids (VSs)^c^	%	54 ± 1.73	1.7 ± 0.14	10.6 ± 0.99
Electrical conductivity (EC)	mS cm^−1^	54.5 ± 0.80	8.36 ± 0.40	24.7 ± 1.20
Salinity	%	27 ± 0.00	4.5 ± 0.70	12.3 ± 0.60
pH	–	8.2 ± 0.02	7.5 ± 0.10	7.5 ± 0.10
Total Kjeldahl Nitrogen (TKN)	mg L^−1^	–	–	3956 ± 3.30
Total Ammonia Nitrogen (TAN)	mg L^−1^	2079 ± 180^b^	1200 ± 0	2462 ± 31
Free Ammonia Nitrogen (FAN)	mg L^−1^	349 ± 0^b^	46 ± 0	179 ± 0

The data is represented as mean of triplicate, with the standard deviation (SD) from the mean.

a Unit is expressed as g g-VS^-^

b Unit is expressed as mg g-VS^−1^.

c Unit is expressed as a percentage of wet weight.

### 2.2 Biochar characterisation and pretreatment

The wood biochar used in this study was sourced from Grayson Australia, Tecnica Pty. Ltd., situated in Melbourne, Australia. This specific wood biochar was made from pyrolysis of hardwood, at 550 °C for a period of 2 h. Prior to use, biochar was pass through a sieve of 0.5–2 mm and rinsed using Milli-Q water to wash out impurities. The biochar was dried in an oven for 2h at 105 °C; this biochar was denoted as BC.

Acid-alkali-treated biochar, designated as treated biochar (TBC), was prepared using HNO3 and NaOH following the method outlined by Ngo, Khudur [5]. Initially, dried wood biochar was immersed in an 8 M HNO_3_ solution at a 1:5 w/v ratio for an 8-h duration. Subsequently, the HNO_3_-treated biochar underwent sieving and rinsed three times using Milli-Q water. The washed HNO_3_-biochar was treated using 0.4 M of NaOH solution at a 1:20 w/v ratio for 24 h. The acid-alkali-treated biochar was thoroughly flushed using Milli-Q water up to a point where constant pH was achieved. Finally, resulting products were heated in an oven at 105 °C for 2 h to yield the treated biochar (TBC).

The surface morphologies of both biochar variants were assessed using FEI Scanning Electron Microscopy (SEM) (Oregon, USA). The images were obtained at a magnification of 5000x, utilising a 5.0 spot size and a voltage of 30.0 kV. A previous research by Ngo, Khudur [5] has extensively characterised the properties of these biochar samples. For the quantification of surface elemental compositions (Na, C, O, and N) of both BC and TBC, both pre-digestion and post-digestion, X-ray photoelectron spectroscopy (XPS) was employed with equipment from Thermo Fisher Scientific in Waltham, MA, USA. The XPS data was analysed using the CasaXPS software program. In addition, Fourier Transformed Infrared (FTIR) spectroscopy was utilised to study surface functional groups of the two biochar types in the wavelength range of 4000 to 650 cm^−1^.

### 2.3 Anaerobic digestion design and destructive sampling

Mixtures of feedstock and inoculant were used in batch-digestion via tightly sealed Schott bottles (250 mL) to determine CH_4_ potential. The digesters were maintained at 37 °C in a water bath using a thermo-regulator. Constant recirculation of water (at 37 °C) water was carried out using a built-in water-pump. The digestion temperature was monitored daily via a built-in thermometer on the thermo-regulator. Trapped biogas collected from the top of the gas cylinders via water displacement was used to determine cumulative gas production (S1). A similar method was used by Kassongo, Shahsavari [22] and Ngo, Khudur [5].

Treatments were performed in triplicate: (i) BC, (ii) TBC and (iii) Control (no biochar). In addition, 3 additional sets of triplicates were set up for each treatment, amounting to a total of 12 replicates per treatment. These triplicates were used in a series of destructive sampling every 10 days to monitor changes in the microbiome of each treatment over time. A total of 5 sampling points were used: day 0, day 10, day 20, day30 and day 40. A treatment containing only the wastewater sludge inoculant was also set up to determine background CH_4_ production.

A substrate to inoculum ratio (SIR) of 7:1 CM to inoculant was used across digesters. A 15 % TS, excluding biochar, was achieved by addition of Milli-Q water. A high substrate to inoculum ratio (SIR) was used to maximise the amount of poultry litter within the digesters. In turn, this would prevent substrate depletion from occurring before the endpoint of 40 days, as well as ensure high levels of total ammonia nitrogen concentrations will be produced to induce ammonia inhibition. The high SIR will also allow for the investigation into biochar's ability to reduce the lag phase, as a high SIR is often associated with longer adaptation time of microbes, resulting in the delay in methane production [23]. Biochar (both variants) was mixed into the digesters using a 1:2 ratio of biochar: CM by dry weight. A working volume of 190 mL was used for each digester. Biochar addition was approximately 5 % of the total working volume, the dosage is close to the ideal range highlighted by Cai, Zhu [19]. Before sealing, nitrogen gas was used to purge the Schott bottles to establish anaerobic conditions. Subsequently, the sealed digesters were subjected to mixing for a duration of one day, at 37 °C using an orbital shaker, to obtain a homogenous mixture. The digestion period lasted 40 days; the captured biogas was gathered and subjected to analysis at 24-h intervals via extraction of the accumulated gas using a syringe (S1). The collected gas was screened for CH_4_ and O_2_ using the MX6 iBrid Portable Multi Gas Monitor, Industrial Scientific (Pittsburgh, Pennsylvania, USA) via built-in infrared sensors.

### 2.4 CH_4_ composition calculation

The daily CH_4_ production was obtained by assessing the total biogas volume collected (tBiogas) and the %CH_4_ composition, analysed with an MX6 iBrid (% CH4). To calibrate further, the daily CH_4_ from the digesters containing only the wastewater sludge inoculant was subtracted. Cumulative CH_4_ production over 40 days was measured as mL g^−1^ VS. The calculations involved are sumarised by the following equation:

Daily CH_4_ = [tBiogas treatment × (% CH_4_)] – [tBiogas inoculant × (% CH_4_)]

Cumulative CH_4_ production was collected at 298 K (room temperature) and adjusted to standard temperature and pressure (STP), which corresponds to 273 K and 1 atm pressure, using the formula Pv = nRT, P = 1 atm, V = mL, n = 1 mol and temperature is in K.

### 2.5 Chemical analysis of digestates

The digestates from day 40 were tested for TAN, pH, COD, salinity and EC following the methods described in Section 2.1. Remaining digestates were frozen at – 18 °C until further required.

### 2.6 DNA extraction

For DNA extraction, an aliquot of 0.25 g from the products of days 10, 20, 30, and 40 (in triplicate) was processed using the DNeasy Powersoil Kit, with the Quick-start Protocol by QIAGEN, based in Hilden, Germany. This procedure involved the use of digestates from all sampling points. Subsequently, the extracted DNA were subjected to absorbance ratios measurements, between 260 nm and 280 nm utilising a NanoDrop Lite Spectrophotometer, Thermo Fisher Scientific (Waltham, MA, USA), for quality and quantity.

### 2.7 Real-time Quantitative-polymerase chain reaction

The DNA extracted from the digestates obtained on days 0 and 40 was subjected to real-time polymerase chain reaction (qPCR), with the QIAGEN Rotor-Gene instrument from Hilden, Germany. This analysis followed an established methodology outlined by Shahsavari, Aburto-Medina [24]. For qPCR amplification of the 16S rRNA, the 341F/518R primer set was employed. Quantifying the 16S rRNA provides valuable insights into the variations in the bacterial biomass. Prior to further analysis, the data obtained from qPCR underwent a log-transformation.

### 2.8 16S amplicon based sequencing for microbial (bacterial and archaeal) community analysis

Using the extracted DNA samples, V4 region of the 16 S rRNA gene was utilised for sequencing. The primers 341F and 806R were used as forward and reverse primers, respectively. All sequencing preparations were conducted following the *Illumina* protocol [25]. Gel electrophoresis and a Bioanalyser 2100 (Agilent, Santa Clara, California, USA) were used for quality assurance testing on PCR products, while a Qubit 4.0 fluorometer (Invitrogen, USA) was used to quantify DNA concentrations. The sequencing data has been recorded in the sequencing read archive database, accessible via PRJNA946321.

### 2.9 Data analysis

All data were presented as means accompanied by standard deviations for the triplicates. Data manipulation was performed using MS Excel. MINITAB-21 software was utilised to conduct One-Way Analysis of Variance (ANOVA). Statistical significance between datasets was examined at at p < 0.05.

For the calculation of microbial diversity indices, rarefied abundances were used. To investigate the effects of biochar supplementation on microbial communities, the phyloseq package was employed to conduct compositional comparisons of bacterial and archaeal abundances within R Studio.

Furthermore, the Analysis of Compositions of Microbiomes with Bias Correction 2 package (ANCOM-BC2) was employed to determine the statistical significance between bacterial and archaeal abundances at a significance level of p < 0.05, with p-values altered by the Holm-Bonferroni method, all within R Studio. ANCOM-BC2 provides these adjusted p-values as part of the outputs to evaluate the significance of differences between microbiome datasets, considering their compositional nature, where statistical differences are determined based on relative abundance rather than absolute abundance.

## 3 Results and discussion

### 3.1 Impacts of BC and TBC addition on cumulative CH_4_ production and digestates

**Fig. 1:**
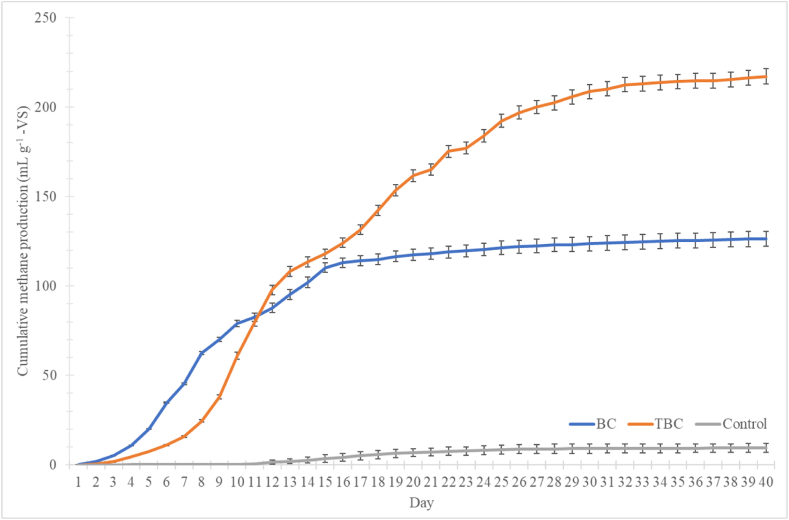
CH_4_ production (cumulative) over 40 days, measured in mL g^−1^ -VS, by biochar (BC, blue) acid-alkali-treated biochar (TBC, orange), and no biochar treatments (Control, grey). The data is represented as the mean of triplicate measurements along with the standard deviation (SD) of the mean.

The impacts of biochars (both variants) on cumulative CH_4_ production over 40 days is shown in Fig. 1. Cumulative CH_4_ production presents different profiles amongst the treatments: a lag phase, an exponential phase, a semi-inhibition phase and an inhibtion phase. The profile differed significantly between treatments (p < 0.05). The Control treatment exhibited the longest lag phase of 11 d compared to the biochar treatments where a lag phase of 1 d was observed. This finding aligns with the results of a previous study where biochar addition, regardless of type, shortened the lag phase significantly compared to the Control treatment [5]. In addition, Hoang, Goldfarb [26] stated that biochar addition can reduce microbial lag-phase by 38 %. Reduction of the lag phase is of economic importance to large-scale anaerobic digesters since it directly translates to higher efficiency of CH_4_ production [27]. The use of biochar in this study, regardless of types, have proven to be an effective agent in minimising the microbial lag phase even under the high SIR. Similar findings have been reported by Masebinu, Akinlabi [28], where the porous structure of biochar can facilitate surface microbial growth and accelerates acclimatisation rate in the event of substrate-induced inhibition.

By day 40, ANOVA revealed Control treatment producing a significantly lower volume of cumulative CH_4_ compared to the BC and TBC treatments, 9.37 compared to 126 and 217 mL g^−1^ -VS, respectively (p < 0.05). A cumulative CH_4_ production of 9.37 mL g^−1^ -VS suggests Control treatments experienced process inhibition by ammonia. The FAN concentration in the Control on day 10 was determined to be 361 mg-FAN L^−1^, which increased to 514, 693 and 714 mg-FAN L^−1^ by day 20, 30 and 40, respectively (S3). This observation aligns with the findings of Peng, Zhang [29], where FAN concentrations above 300 mg L^−1^ resulted in process inhibition. The addition of BC and TBC led to an increase in cumulative CH_4_ production over the Control. These findings align with those reported in several other studies where a significant improvement in cumulative CH_4_ production was observed following the addition of biochar additives [18,[30], [31], [32]]. In addition, cumulative CH_4_ production for TBC was 1.7 times higher then BC treatments on the 40th day (p > 0.05). Digesters amended with BC experienced inhibition from around day 16, where gas production was less than 1 % of the total production. Song, Qiao [33] reported a typical methane potential range for the AD of CM between 250 and 450 mL g^−1^ -VS; only the use of TBC could produce a methane production close to this range.

TS destruction was significantly higher for BC and TBC compared to the Control according to ANOVA; 32 % and 47 % compared to 5 %, respectively (p < 0.05) (Table 2). Similarly, VS destruction was significantly improved for BC and TBC in comparison to the Control; 27 % and 46 % compared to 5 %, respectively (p < 0.05) (Table 2). These observations further confirm the significant increase in the utilisation of the organic fraction by digesters treated with BC and TBC, leading to higher cumulative methane production. In addition, the significantly higher TS and VS removal by TBC compared to BC also relates to its higher cumulative methane production (p < 0.05).

**Table 2:** Characteristics of the day 40 digestates and their percentage variation in comparison to the starting (d0) material.

Characteristics	Control		BC treatment		TBC treatment	
	Values	% Δ	Values	% Δ	Values	% Δ
Total COD (g L^−1^)	10.16 ± 0.13	−5	8.54 ± 0.22	−20	7.91 ± 0.21	−26
Electrical conductivity (mS cm^−1^)	27.8 ± 1.81	+11	17.8 ± 0.31	−29	20.5 ± 0.48	−18
Salinity (%)	14 ± 1.00	+14	8.33 ± 1.54	−32	10.7 ± 0.58	−14
pH	8.24 ± 0.23	N/A	7.84 ± 0.22	N/A	8.01 ± 0.28	N/A
TS (%)^a^	13.9 ± 0.92	−5	9.85 0.21	−32	7.65 ± 0.92	−47
VS (%)^a^	11.1 ± 0.35	−5	8.45 ± 0.21	−27.	6.24 ± 0.64	−46

Values, excluding the % change, are represented as the mean of triplicate measurements along with the standard deviation (SD) of the mean.

N/A: not applicable.

a Unit is expressed as % of wet weight.

The exponential phases differed between treatments; BC treatment showed an exponential phase lasting 15 days, while the TBC treatment showed an exponential phase lasting 31 days. Both exponential phases of BC and TBC treatments showed steep curves, indicative of rapid substrate degradation. In contrast, the Control treatment did not show a clear exponential phase, suggesting complete NH_3_ inhibition within the Control digesters. This was also reflected in the lower level of TS and VS destruction observed in the Control treatment (Table 2). In addition, the electrical conductivity (EC) of Control treatments increased by 11 % (Table 2). This was most likely due to the unmediated release of NH_4_^+^ within the reactors, EC did not increase for BC and TBC treatments [5,34].

Furthermore, the reduction of COD after 40 days of AD in the Control treatment was only 5 %, compared to the 20 % and 26 % of BC and TBC treatment. This suggests a significant build-up of organic matter and inhibition (Table 2). The higher cumulative methane production observed in TBC treatment could have been a result of better mitigation against NH_3_ stress for the microbial communities. This may have promoted more microbial growth within TBC digesters compared to that of the BC digesters, potentially leading to a significantly higher degree of TS and VS destruction (p < 0.05) as determined by ANOVA (Table 2). The underlying mechanisms leading to this observation will be discussed below.

### 3.2 Biochar and TAN removal

#### 3.2.1 Total ammonia nitrogen concentration and removal efficiency demonstrated by biochar and treated biochar

Total Ammonia Nitrogen increased in Control and BC treatments, but decreased in the TBC treatment by the 40th day, relative to the TAN concentration at day 0. The Control treatment showed the highest spike in TAN, with a 60.1 % increase, followed by a 10.3 % increase in the BC treatment (Table 3). In contrast, the use of TBC produced a 6.8 % decrease in TAN on day 40. By day 40, ANOVA showed the TAN concentrations of 3 treatments differing significantly (p < 0.05). This suggests that the addition of both biochar variants to adsorb NH_4_^+^ was successful compared to Control.

**Table 3:** The initial (d0) and final (d40) measurements of TAN, FAN concentration, and changes in TAN concentration and percentages.

Treatment	Day 0 TAN (mg-TAN L^−1^)	Day 40 TAN (mg-TAN L^−1^)	d_40_ FAN (mg-FAN L^−1^)	ΔTAN^a^ (mg-TAN L^−1^)	ΔTAN (%)
Control	2400 ± 31	3920 ± 195	714 ± 0	+1458	+60
BC treatment	2400 ± 31	2657 ± 258	218 ± 0	+257	+10
TBC treatment	2400 ± 31	2311 ± 62	270 ± 0	−169	−7

Values, excluding the % change, are represented as the mean of triplicate measurements along with the standard deviation (SD) of the mean.

a Difference between day_0_ and day_40_.

These observations are similar to those found in the literature, where the use of biochar was effective in reducing TAN concentrations via NH_4_^+^ adsorption [18,20,31,35,36]. This significant reduction in TAN in the biochar treatment leads to improvements in CH_4_ production kinetics compared to that of the Control. Digesters treated with BC and TBC also experienced lower levels of NH_3_ stress from FAN; the FAN concentrations for BC was 218 mg-FAN L^−1^, and TBC was 270 mg-FAN L^−1^, respectively. This was approximately 3.5 times less than that of the Control digester, at 714 mg-FAN L^−1^. A study by Zhang, Yuan [37] found that 337 mg-FAN L^−1^ decreased methane yield by 65 %. This aligns with the findings of this study where a significant reduction in methane yield was experienced in Control digesters (Fig. 1). The results suggest that biochar addition in high solids AD of CM presents a cost-effective and efficient additive to mitigate the build-up of TAN, FAN and prevent NH_3_ inhibition. This aligns with findings of the current literature, where addition of biochar can adsorb TAN and produce positive impacts on NH_3_ stressed AD systems [19].

The use of TBC produced a significantly stronger adsorption effect than BC (p < 0.05). On day 40, the TBC treatment achieved a 29 % improvement in TAN removal efficiency compared to the BC treatment. The findings of this study show that the use of acid and alkali treatment on biochar can significantly improve NH_4_^+^ adsorption, as previously reported [5,20]. The mechanisms leading to this increase in adsorption capacity will be discussed further below.

#### 3.2.2 Biochar characteristics and mechanisms of TAN removal

**Fig. 2:**
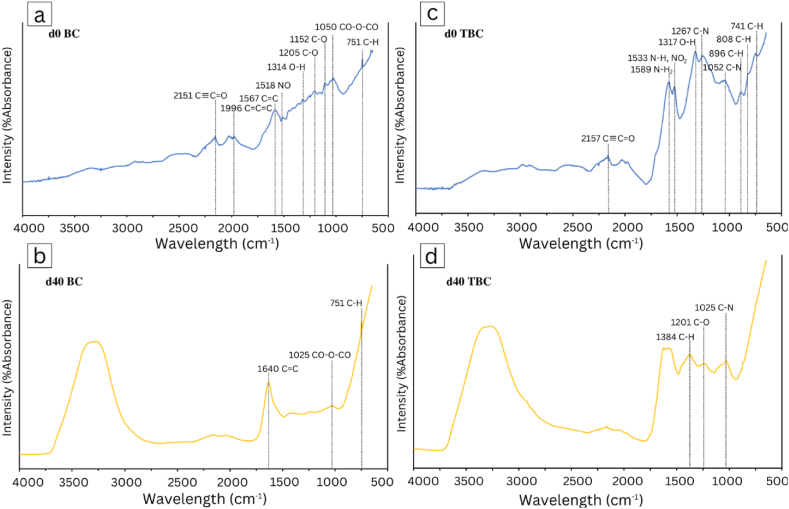
Fourier Transformed Infrared (FTIR) spectra of d0 BC (a), d40 BC (b), d0 TBC (c) and d40 TBC (d).

Both FTIR and XPPS were employed on day 0 and day 40 biochar samples taken from BC and TBC treatments to investigate chemical changes that had occurred during acid-alkali treatment as well as the AD process. These changes are outlined in the following Fig. 2 and Tables 4a and 4b.

**Table 4a:** FTIR spectra and X-ray photoelectron spectroscopy (XPS) results from d0 BC, d0 TBC, d40 BC and d40 TBC.

Waveband (cm^−1^)	Functional groups
**2151–2157**	C=C=O, ketene
**1900–2000**	C=C=C, allene
**1600–1650**	C=C stretching, conjugated alkene
**1566–1650**	C=C stretching, cyclic alkene
**1543–1670**	NH_2_ groups
**1543–1470**	NH, NO_2_ groups
**1310–1390**	O–H, phenol
**1200–1225**	C–O stretch, vinyl ether
**1125–1205**	C–O stretch, tertiary alcohol
**1040–1050**	CO–*O*–CO stretching, anhydride
**1020–1250**	C–N stretching, amine
**860–900**	C–H bending, 1&3-disubstituted, 1,2&4-trisubstituted
**790–830**	C–H bending, 1,2,3,4-tetrasubstituted, 1&4-disubstituted
**730–770**	C–H bending, monosubstituted

**Table 4b:** Atomic composition of Carbon, Nitrogen, Oxygen and Sodium from X-ray photoelectron spectroscopy (XPS) analysis, in percentage (%), for d0 BC, d0 TBC, d40 BC and d40 TBC.

Atomic composition (%)	d0 BC	d0 TBC	d40 BC	d40 TBC
Sodium	0.00	2.23	0.00	0.00
Carbon	75.87	74.51	72.54	70.89
Nitrogen	0.71	2.79	4.40	7.01
Oxygen	23.52	20.47	23.06	22.1

Based on Fig. 2, the acid-alkali treatment of BC yielded changes in functional groups mainly in the form of N incorporation. Table 4a also highlights the various functional groups found on BC and TBC, before and after digestion. Moreover, the treatment with acid and alkali resulted in the loss of the C=C=C and C=C functional groups at wavebands 1900–2000 and 1566 - 1650 cm^−1^; TBC did not exhibit these peaks (2c). This observation has been previously documented by Jin, Sun [38] and Chang, Tian [39], where the use of HNO_3_ resulted in oxidative cleaving of carbon bonds within biochar particles. Secondly, the use of HNO_3_ resulted in the formation of new C–H groups at the wavebands 860–900 and 790–830 cm^−1^. Further, HNO_3_ treatment resulted in the formation of new N-containing groups such as NH_2_, NH, NO_2_ and C–N at wavebands 1543 - 1670 cm^−1^, 1543–1470 cm^−1^ and 1020–1250 cm^−1^, respectively. These new peaks observed in TBC are consistent with the findings of several studies where the use of HNO_3_ produced more N-containing functional groups that may increase the redox potential of the biochar [5,[38], [39], [40]].

The relative surface atomic composition (%) of carbon, nitrogen, oxygen and sodium, for both BC and TBC, is shown in Table 4b. The results from XPS further confirmed that treatment of BC with HNO_3_ incorporated nitrogen groups onto the biochar surface, with nitrogen bonding increasing from 0.71 % to 2.79 % when comparing d0 BC and d0 TBC, respectively. Lang, Yan [41], Gao, Wang [40] and Wang, Li [42] also reported the introduction of surface nitrogen groups onto biochar treated with HNO_3_. Sodium bonding also increased following NaOH treatment, from 0 % to 2.23 % when comparing between day 0 BC and day 0 TBC. This increase in surface sodium has also been reported by Vu, Trinh [20] and can significantly improve TBC's adsorption capacity towards NH_4_^+^. This increase in surface sodium can result from the formation of salt forms from the acidic form when HNO_3_-treated biochar was soaked in NaOH.

At day 40, both BC and TBC exhibited changes in functional groups. Overall, there appeared to be a decrease in functional groups on day 40 BC and TBC compared to day 0 BC and TBC. Both BC and TBC also exhibited an intense peak between the 3700-3200 cm^−1^ waveband belonging to the –OH group (2b, 2d). However, this was due to the recovered biochar's wet nature before their analyses. On day 40, the BC FTIR spectrum showed a loss of various groups: O–H, C–O, and CO–O–CO at 1310–1390 cm^−1^, 1200–1225 cm^−1^, 1125–1205 cm^−1^ and 1040–1050 cm^−1^ waveband, respectively (Fig. 2a and b). This suggests that BC's main mechanism for NH_4_^+^ adsorption was electrostatic attraction; NH_4_^+^ react with surface OH^−^ or COO^−^ groups (Ngo et al., 2022). The FTIR spectrum of TBC showed a loss of functional groups: the O–H and C–H groups at the 1310–1390 cm^−1^, 860–900 cm^−1^ and 790–830 cm^−1^ wavebands, respectively (2d). While the loss of O–H suggests electrostatic attraction similar to that of BC, the loss of C–H is indicative that the main mechanism for NH_4_^+^ adsorption by TBC is complexation via van der Waals forces [43]. Complexation is a weak chemical bond that does not require high activation energy compared to other types of chemical bonds and therefore, can react much faster leading to higher NH_4_^+^ adsorption by TBC as discussed in Section 3.2.1 [44]. Moreover, XPS results further confirmed a higher level of surface nitrogen bonding by TBC compared to BC on day 40, 7.01 % compared to 4.4 %, respectively. In addition, the surface sodium of TBC decreased from 2.23 % at day 0 to 0 % at day 40, suggesting that TBC also exhibited cation exchange to adsorb NH_4_^+^, losing surface Na^+^ in the process.

The results from FTIR and XPS suggest that the use of HNO_3_ and NaOH treatment was effective in improving the NH_4_^+^ adsorption capacity. Acid treatment of BC resulted in an increase in C–H groups observed in TBC which in turn, promoted rapid surface complexation of NH_4_^+^. Alkali treatment also introduced surface sodium onto TBC particles, which promoted cation exchange to adsorb NH_4_^+^, this mechanism was not present in BC particles. These observations may explain to the higher level of TAN removal observed in TBC compared to BC. Consequently, they have translated to better CH_4_ production kinetics observed in biochar treatments compared to the Control in Fig. 1. This reduction in TAN may have increased the resilience of ammonia sensitive microbes within these digesters.

### 3.3 Change in biochar surface Morphology over time

Scanning Electron Microscopy was used to monitor microbial interactions with BC and TBC over the span of the AD process. Images were taken for both biochar type at day 10, 20, 30 and 40. Clear morphological differences exist between the two biochar types. A previous study reported that BC possesses a rough, flaky surface with pores that are poorly developed, while TBC shows a rough structure with well-developed macropores (>50 nm) [5]. The use of HNO_3_ treatment corrodes the organic structure of the biochar, volatilising certain organic compounds and improving the abundance and size of pores [45].

**Fig. 3:**
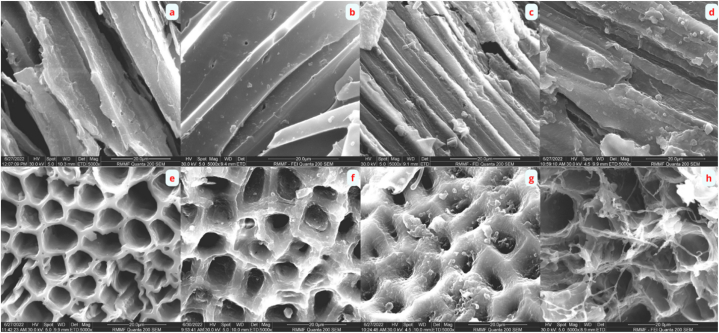
SEM images of wood biochar on d10 (a), d20 (b), d30 (c) d40 (d) and treated-wood biochar on d10 (e), d20 (f), d30 (g) and d40 (h). Images were taken at 5000x magnification, 30.0 kV and 5.0 spot size.

Fig. 3a–d shows the changes in microbial attachment on the surface of BC from day 10, 20, 30 and 40, respectively. Small-sized microbes of around 1–2 μm were detected on the surface of BC from day 20 onwards (Fig. 3d). This observation aligns with the findings of Wang, Zhang [46], where it was reported that small-sized microbes are generally enriched in biochar possessing smaller pore volume. No large-sized microbes were detected on the surface of BC throughout the 40 days of AD.

Fig. 3e–h shows the changes in microbial attachment on the surface of TBC from day 10, 20, 30 and 40, respectively. Starting from day 20, small-sized microbes were detected within the pores of TBC. On day 30, SEM images showed clusters of irregularly shaped coccoid cells proliferating within the pores of TBC, an SEM image taken at 10,000x magnification further confirms this (S2). This may indicate the presence of members of the family Methanosarcinaceae*,* which are known to exist in aggregates consisting of large numbers of cells under stressed conditions such as high NH_3_ concentrations [47]. On day 40, the surface of TBC appeared to be highly colonised by long, filamentous microbes. This may suggest the presence of members of the family Methanosaetaceae, whose growth physiology have been well documented to be long and filamentous [48,49]. Zabranska and Pokorna [47] also reported that filamentous methanogens in the family Methanosaetaceae are extremely sensitive to NH_3_ and hence, their presence observed in Fig. 3 suggests that TBC exhibited a stronger microbial sheltering effect.

The acid-alkali treatment created a rougher and more porous biochar surface, allowing TBC to exhibit a stronger microbial sheltering effect. This observation has been previously reported by Johnravindar, Kaur [50], where surface roughness and degree of porosity have direct effects on microbial attachment and proliferation. Similarly, Cai, Zhu [19] also reported that biochar porosity can affect the growth and survival of microorganisms, only macropores can benefit microbial colonisation. While the use of both BC and TBC greatly improved the efficiency of the AD system and mitigated well against NH_3_ stress, the increase in microbial sheltering and biofilm formation observed lead to a significant improvement in CH_4_ production between TBC and BC (Fig. 1). These observations may carry further implications for future studies, specifically for semi-continuous or continuous AD systems. The changes in microbial communities and their interactions with biochar particles will be further explored below.

### 3.4 The impacts of biochar and treated biochar on bacterial and archaeal relative abundance and structure

#### 3.4.1 Total microbial population analysis

**Fig. 4:**
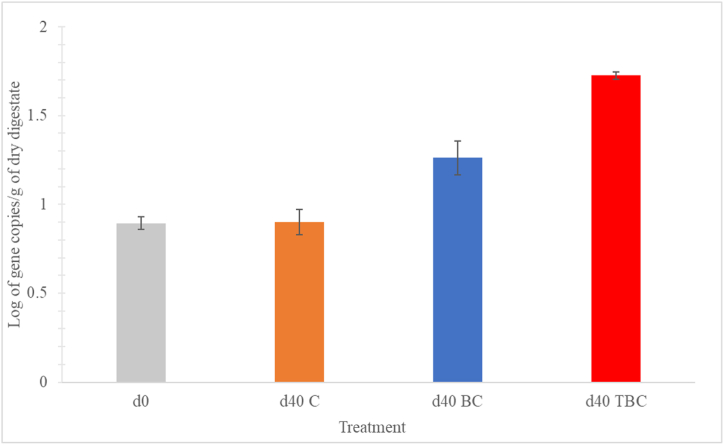
Alterations in microbial biomass (evaluated as log_10_ 16S rRNA gene copies) per gram of dry digestates in different treatment groups: Control, BC and TBC, for day 0 and day 40. The data is represented as the mean of triplicate measurements along with the standard deviation (SD) of the mean, represented by error bars.

The qPCR results indicated variances in the quantity of 16S rRNA gene copies following 40 days of anaerobic digestion (AD); the microbial biomass differed significantly across all treatments, according to ANOVA (p < 0.05) (Fig. 4). Overall, microbial biomass increased with biochar addition. In contrast, microbial biomass remained unchanged in the Control treatment over time. This can be attributed to NH_3_ inhibition during AD.

When comparing between day 40 digestates of the 3 treatments, TBC treatment showed a significantly higher number of gene copies compared to the other treatments (p < 0.05). The number of gene copies were 2.9 and 6.7 times higher in TBC compared to BC and Control on day 40, respectively. The BC treatment also produced a significant higher number of gene copies than the Control treatment (p < 0.05). Between Control and day 0, no significant differences were found, suggesting that microbial growth was severely inhibited by the high FAN concentrations (p > 0.05)(Table 3). This observation aligns with the conclusions from a prior study, where the addition of TBC was best for preserving microbial communities in an environment of high NH_3_ [5]. The results suggests that 10.13039/100012477TBC exhibited a stronger microbial sheltering effect, this is also supported by the SEM images from Section 3.3. Previous studies have also highlighted the positive effects which that biochar can produce towards microbial acclimation in NH_3_-stressed environments [[51], [52], [53], [54]]. This higher microbial growth observed in TBC digesters translated into a higher cumulative methane production compared to BC digesters (Fig. 1). In addition, the lack of microbial growth observed in Control treatments align with its low methane production, which was completely inhibited.

#### 3.4.2 Microbial structure and abundance analysis

**Fig. 5:**
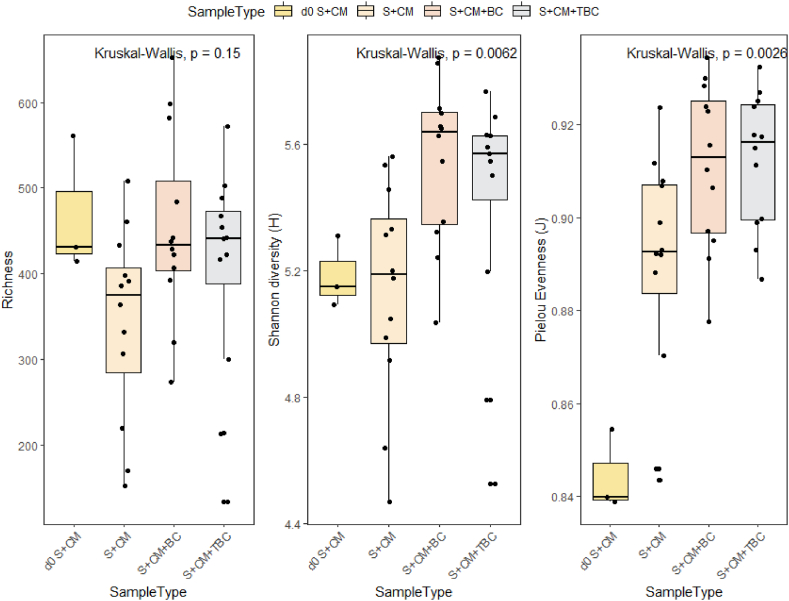
The effect of control treatment (S + CM), wood biochar treatment (S + CM + BC) and treated biochar treatment (S + CM + TBC) on Richness, Shannon Diversity and Pielou Evenness indices of the microbial communities obtained from the day 40 digestates, in comparison to that of the day 0 feedstock (d0 S + CM).

To further investigate the response of microbial populations within anaerobic digesters amended with BC and TBC, the day 0 feedstock and digestates from different sampling points were used for microbial community analysis using 16S amplicon-based sequencing. Fig. 5 shows the Richness, Shannon diversity and Pielou Evenness indices for the day 0 feedstock and the day 40 digestates.

Microbial richness remained relatively unchanged throughout the 40 days for BC and TBC treatments, while the Control treatment showed a decrease in microbial richness. In addition, the use of both biochar treatments produced an increase in Shannon diversity. In contrast, microbial diversity remained relatively unchanged after 40 days in the Control digesters. Similarly, Faisal, Ebaid [55], Di, Zhang [56] and Guo, Jalalah [57] highlighted that AD treated with biochar resulted in higher richness and diversity compared to untreated digesters. A possible explanation for the increase in richness and diversity is the ability of biochar to adsorb contaminants and function as a microbial carrier [57,58].

Additionally, microbial community evenness was assessed using the Pielou Evenness index. Over the 40 days of AD, Pielou Evenness increased in all treatments. This indicated that some bacterial taxa had proliferated and grown increasingly dominant throughout the AD process, while others were reduced in relative abundance.

#### 3.4.3 Archaeal community diversification from biochar and treated biochar addition

**Fig. 6:**
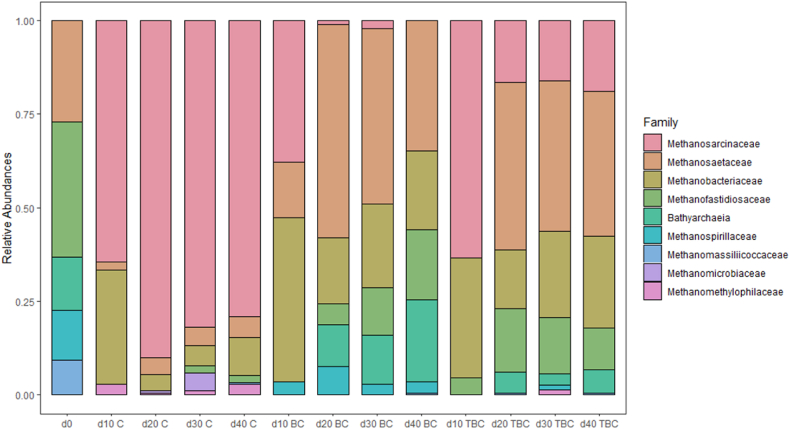
The effect treatments on the relative abundance of archaeal family measured over the 5 sampling points (d0, d10, d20, d30, d40). Values represent the mean of three replicates without error bars. Abundance and legend bars are organised by mean family abundances. Only 9 archaeal families were identified.

Microbial community dynamics under different treatment conditions were investigated and the findings summarised in Fig. 6. To gain insight into the shift of microbial communities over time, bacterial phyla and archaeal families were measured at day 0 and at each sampling point from each treatment. As the focus of the study was on archaeal community diversification, only data for archaeal families is presented, as relative abundance, in Fig. 6. The changes in bacterial phyla has been included as a supplementary figure (S4).

The observations from the bacterial community analysis suggests that biochar addition of both variants did not produce changes over time when compared to the Control; structural variation of the bacterial communties were consistent across all treatments. While ANCOM-BC2 showed that biochar addition did not produce significant changes in the bacterial communities (p > 0.05), the results indicate a healthy bacterial community across all treatments. The bacterial phyla with the highest abundance in the AD of CM were Bacteroidota and Firmicutes; this was consistent throughout all digesters at various sampling points. Fermentative bacteria belonging to these phyla are often dominant within anaerobic digesters [59,60]. Bacteroidota has been known to participate in the decomposition of polysaccharides such as cellulose, which is a component of CM [59]. Similarly, the increase in the relative abundance of Firmicutes is desirable since these bacteria are crucial for hydrolysing various organic substrates, such as proteins, lipids, and polysaccharides, all of which are found in CM [50,61]. The high relative abundance of Bacteroidota and Firmicutes is desirable as these phyla are key players in hydrolysis, a key step within the AD process (S4). Compared to the bacterial phyla, the addition of biochar may have produced more desirable effects on the methanogenic population over time. This will be further discussed in the following section.

At day 0, the most abundant archaeal family was *Methanofastidiosaceae*, accounting for 36 % of the relative abundance; *Methanosaetaceae* had the second highest relative abundance of 27.1 % (Fig. 6). *Bathyarchaeia*, *Methanospirillaceae* and *Methanomassiliicoccaceae* were also identified at 14.1 %, 13.3 % and 9.3 %, respectively.

At day 10, ANCOM-BC2 showed that the relative abundance of *Methanofastidiosaceae* was significantly reduced across all digesters (p < 0.05). In Control and BC digesters the relative abundance of *Methanofastidiosaceae*'s was <1 %. In contrast *Methanofastidiosaceae*'s relative abundance in TBC digesters was 11 % at day 10. According to Walter, Hanser [62] and Nobu, Narihiro [63], *Methanofastidiosaceae* species do not metabolise acetate and produce CH_4_ solely via the demethylation of methylated thiols. Hence, *Methanofastidiosaceae* tend to dominate in low acetate environments that hamper the growth of other acetoclastic methanogens. This suggests that at day 0, the digesters contained low concentrations of acetate due to a lack of substrate. At day 10, the increase in the concentration of acetate from the breakdown of chicken manure might have indirectly contributed to the rapid decline in the *Methanofastidiosaceae* family. Other acetoclastic methanogens may have then proliferated and gained dominance within the digesters, such as those belonging to the *Methanosarcinaceae* family. In contrast to *Methanofastidiosaceae*, the growth of *Methanosarcinaceae* is favoured in high acetate environments [50,59]. The relative abundance of *Methanosarcinaceae* increased from <1 % at day 0, to 61 %, 37.8 % and 63 % in Control, BC and TBC digesters, respectively. *Methanosarcinaceae* have also been reported to be tolerant to elevated NH_3_ concentrations. This is because they exist mainly in thick clumps of cells, reducing their surface area exposed to free diffusion of NH_3_ [50,[64], [65], [66]]. This microbial shield allows species within the *Methanosarcinaceae* family to better adapt to deteriorating AD conditions, such as NH_3_ accumulation in Control digesters. Another possible explanation for the high relative abundance of *Methanosarcinaceae* observed in BC and TBC after 10 days could be due to biochar addition. As discussed in Section 3.3, BC and TBC provided anchorage points for *Methanosarcinaceae*, allowing increased microbial growth. This finding aligns with that of Li, Sun [59], who reported biochar supplementation has also enriched *Methanosarcinaceae*.

Similarly, the *Methanosaetaceae* family with the second highest relative abundance of 27.1 % in day 0 digesters, experienced a rapid population decline by day 10, down to 2 %, 14.2 % and <1 % in Control, BC and TBC digesters, respectively. Species with the *Methanosaetaceae* family are extremely sensitive to inhibitors such as NH_3_. This is due to their thin, filamentous shape which enlarges their surface area to free diffusion of NH_3_ [47,48,61]. During the early phases of AD, the rapid increase in TAN and FAN might have resulted in the rapid decline of *Methanosaetaceae* family. In contrast, ANCOM-BC2 showed that the family *Methanobacteriaceae* increased significantly from <1 % in relative abundance in day 0 feedstock to 30.4 %, 43.8 %, 31.8 % in Control, BC and TBC digesters by day 10, respectively (p < 0.05). Species belonging to this family have been documented to be resistant to NH_3_, which allowed them to become increasingly dominant [61].

From day 10 onwards, the digesters showed different profiles of archaeal community shifts. In Control digesters, *Methanosarcinaceae* became the most dominant family with 80 % relative abundance on Day 40. Other studies at both full-scale and lab-scale, have also reported *Methanosarcinaceae* as the main acetoclastic family at high NH_3_ levels [67,68]. However, this increase in their relative abundance did improve gas production due to a lack of microbial growth (Fig. 4). The *Methanosaetaceae* family in Control digesters could not recover over time, suggesting their growth to be hampered by ammonia inhibition.

For BC and TBC digesters, the relative abundance of *Methanosarcinaceae* decreased gradually over time. By day 40, the BC digesters had <1 % *Methanosarcinaceae* while in TBC digesters they had a relative abundance of 18.7 %; the difference was shown to be significant, using ANCOM-BC2 (p < 0.05). Both BC and TBC treatments resulted in NH_3_ stress mitigation, as discussed in Section 3.2. This removal of TAN from the system over time allowed other NH_3_-sensitive methanogens to compete with the *Methanosarcinaceae* family. Notably, members of the *Methanosaetaceae* were enriched via the addition of BC and TBC. Their relative abundance increased from 14.8 % to 34.7 % in BC digesters, and from <1 % to 38.7 % in TBC digesters. The higher relative abundance of the *Methanosarcinaceae* family in TBC digesters compared to BC digesters at day 40 could have been a result of the stronger microbial sheltering effect by the more porous TBC particles [19]. While the *Methanobacteriaceae* family decreased over time, they remained in relatively high abundance at day 40 for all digesters, 10 %, 21.1 % and 24.5 %, for Control, BC and TBC digesters, respectively. Lastly, the *Methanofastidiosaceae* family increased gradually in relative abundance over time in BC and TBC digesters. This resurgence of the *Methanofastidiosaceae* family could be due to a decrease in acetate concentration within the digesters over time [62,63].

The results indicate supplementation of biochar to the AD of CM can enrich certain methanogens such as the *Methanosaetaceae* and *Methanosarcinaceae* family via microbial sheltering, as in the case of TBC. In addition, biochar can also indirectly enrich the members of *Methanosaetaceae* family via removal of TAN, as in the case for both BC and TBC. It was found that a combination of TAN adsorption and microbial sheltering was most effective in enriching NH_3_-sensitive methanogens. Biochar addition of both variants also promoted the growth of several other methanogenic families such as *Methanobacteriaceae* by providing anchorage point for surface colonisation and proliferation. This created a more robust archaeal community over time. This increased robustness ultimately translated to higher production of CH_4_. In addition, the preservation of the *Methanosarcinaceae* family in TBC digesters, as well as the higher microbial population observed in Fig. 4, increased the methane yield in comparison to BC digesters. Members of the *Methanosarcinaceae* family are the only methanogens capable of utilising up to 9 different substrates, further improving the robustness of TBC digesters [47].

## 4 Conclusions

The use of biochar significantly reduced TAN levels in digesters and increased CH_4_ production compared to Control digesters. For digesters under NH_3_ stress without biochar addition, microbial growth was inhibited and the archaeal community was dominated by the *Methanosarcinaceae* family. Biochar addition created a more diverse and robust archaeal community which lead to a higher CH_4_ production. Treated biochar produced a stronger microbial sheltering effect, promoting the highest microbial growth and enriching both the NH_3_-sensitive *Methanosaetaceae* and *Methanosarcinaceae* families, while untreated biochar could not preserve the *Methanosarcinaceae* family over time. In this study, only the use of acid-alkali treated biochar could achieve a cumulative methane production close to typical methane potential range for chicken manure operated under optimal conditions. The findings of this study showed that HNO_3_–NaOH treatment of biochar could be an effective method to enhance its performance in AD. In semi-continuous or continuous AD using CM, where a build-up of NH_3_ is inevitable, the effects of TBC on ammonia stress mitigation and archaeal communities will be more pronounced. The use of TBC can be a cost-effective method to aid in the recovery of ammonia inhibited digesters.

## Data availability statement

The sequencing data associated with this study has been deposited into a publicly available repository. The data was deposited into sequencing read archive (SRA) database and can be accessed via the accession number PRJNA946321.

## CRediT authorship contribution statement

**Tien Ngo:** Conceptualization, Data curation, Formal analysis, Investigation, Methodology, Writing – original draft, Writing – review & editing. **Leadin S. Khudur:** Data curation, Project administration, Resources, Software, Validation, Writing – review & editing. **Christian Krohn:** Methodology, Project administration, Resources, Validation, Visualization, Writing – review & editing. **Soulayma Hassan:** Investigation, Methodology, Resources, Writing - review & editing. **Kraiwut Jansriphibul:** Data curation, Methodology, Software, Validation. **Ibrahim Gbolahan Hakeem:** Investigation, Methodology, Resources, Validation, Visualization, Writing – review & editing. **Kalpit Shah:** Conceptualization, Funding acquisition, Investigation, Supervision, Validation, Writing – review & editing. **Aravind Surapaneni:** Conceptualization, Investigation, Methodology, Supervision, Validation, Writing – review & editing. **Andrew S. Ball:** Conceptualization, Funding acquisition, Investigation, Project administration, Resources, Supervision, Validation, Writing – review & editing.

## Declaration of competing interest

The authors declare that they have no known competing financial interests or personal relationships that could have appeared to influence the work reported in this paper.
