# Supplementary material for: Wood biochar enhances methanogenesis in the anaerobic digestion of chicken manure under ammonia inhibition conditions
Source: Heliyon. 2023 Oct 21;9(11):e21100. doi: 10.1016/j.heliyon.2023.e21100 (PMC10618790; doi:10.1016/j.heliyon.2023.e21100)
Supplement: Multimedia component 1 [file mmc1.docx]

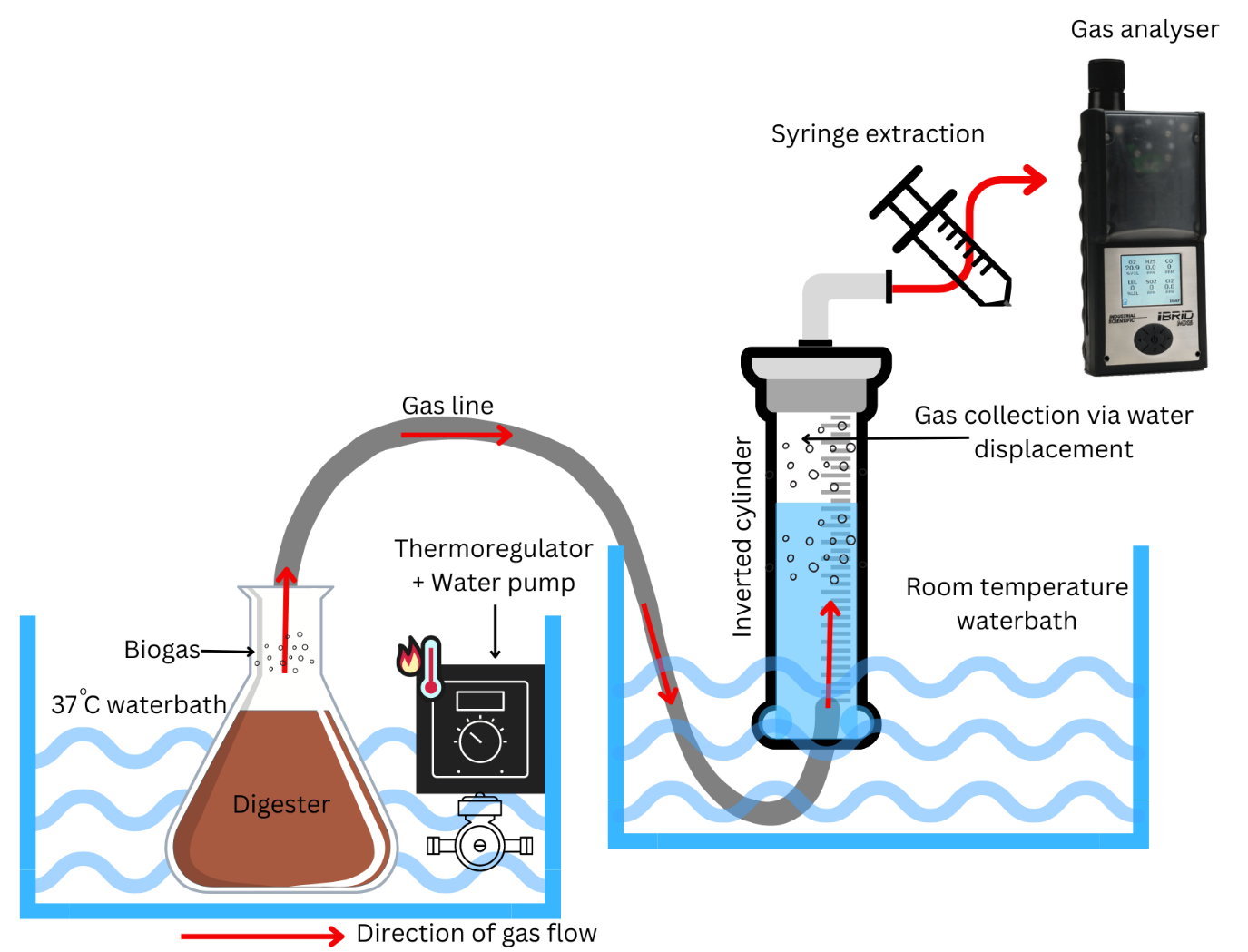


**S1:** Flow diagram of experimental set-up and gas sampling procedure.


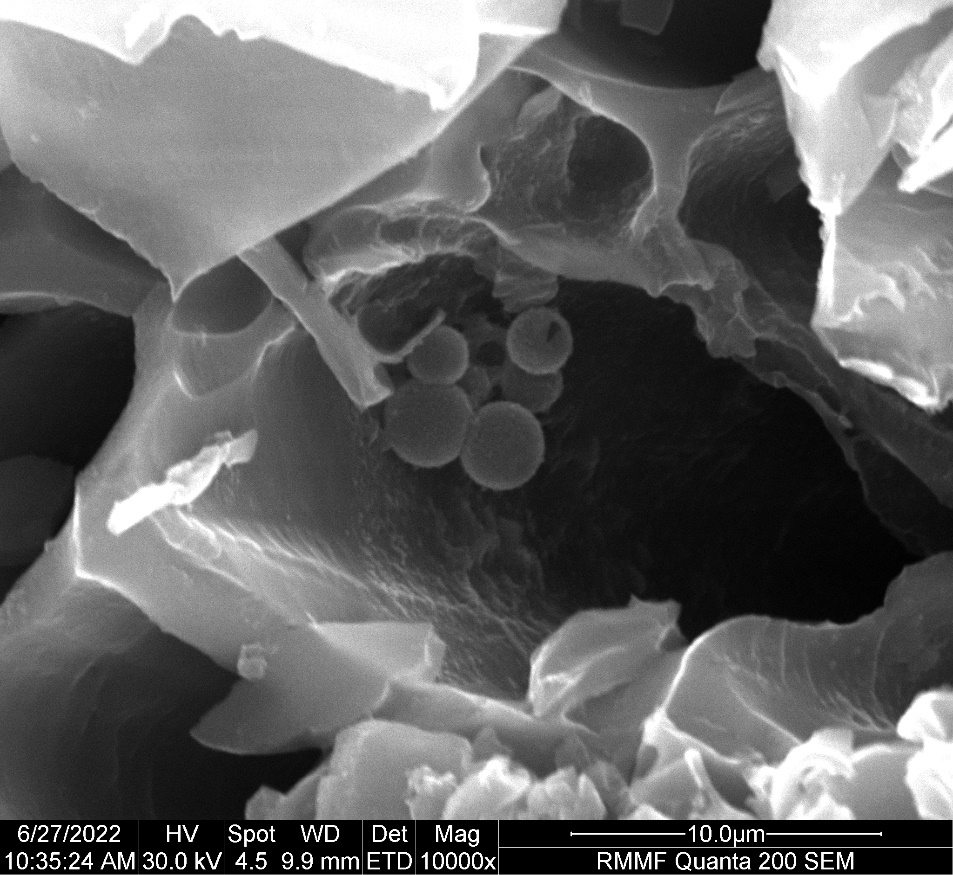


**S2:** SEM image of Treated Wood Biochar (TBC) on day 30, taken at 10,000x magnification, 30.0kV and 5.0 spot size.

**S3:** Changes in pH, TAN and FAN concentration in day 0 feedstock and day 10,20,30 and 40 digestate for Control (C), Biochar (BC) and Treated biochar (TBC).

| **Parameters** | **pH** | **TAN (mg-TAN L^-1^)** | **FAN (mg-FAN L^-1^)** |
| --- | --- | --- | --- |
| d0 | 7.79 | 2462 | 179 |
| d10 C | 8.02 | 3066 | 361 |
| d20 C | 8.11 | 3626 | 514 |
| d30 C | 8.24 | 3804 | 693 |
| d40 C | 8.24 | 3920 | 714 |
| d10 BC | 7.97 | 1866 | 179 |
| d20 BC | 8.17 | 2097 | 220 |
| d30 BC | 8.12 | 2560 | 254 |
| d40 BC | 8.02 | 2657 | 218 |
| d10 TBC | 7.97 | 1973 | 208 |
| d20 TBC | 8.17 | 2026 | 319 |
| d30 TBC | 8.12 | 1973 | 285 |
| d40 TBC | 8.02 | 2311 | 270 |


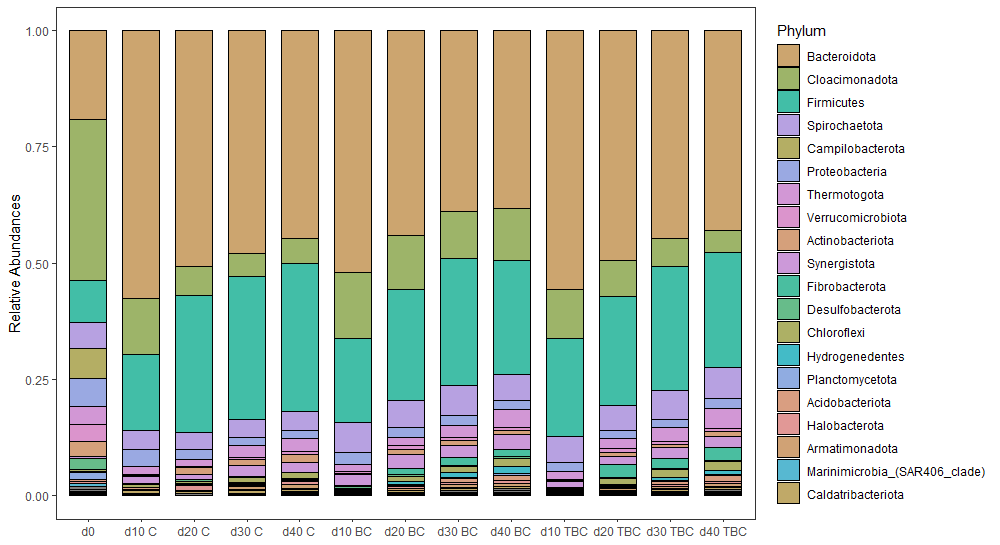


**S4:** The effect of control treatment (No biochar), wood biochar treatment (BC) and treated biochar treatment (TBC) on the relative abundance of bacterial phyla measured over the 5 sampling points (d0, d10, d20, d30, d40). Values represent the mean of three replicates without error bars. Abundance and legend bars are organised by mean phylum abundances. Only the top 20 bacterial phyla are shown.
